# Whitlow

**Published:** 1910-04-30

**Authors:** F. C. Pybus

**Affiliations:** late Surgeon to Out-patients' Dressing Department, Royal Victoria Infirmary, Newcastle-on-Tyne.


### The General Practitioner's Column.

[Contributions to this Column are invited, and, if accepted, will be paid for.]

### WHITLOW.

By F. C. PYBUS, M.B., B.S., F.R.C.S.; late Surgeon to Out-patients' Dressing Department, Royal Victoria Infirmary, Newcastle-on-Tyne.

### II.—COMPLICATIONS AND TREATMENT.

The commoner complications of whitlow may be classified as follows:—

| | |
|---|---|
| Suppuration in the palm. / ,, ,, forearm. | Superficial or Deep. |
| Lymphangitis. | Lymphadenitis. |

Suppuration in the subcutaneous tissue of the finger may spread towards the hand, causing pus formation superficially, chiefly at the roots of the fingers, in the spaces between the heads of the metacarpal bones. Abscesses may also form under the skin of the palm, or more deeply under the palmar fascia and yet not invade the synovial sheath. Thecal suppuration, especially if involving the thumb or little finger, readily spreads up the synovial sheaths into the palm. That form occurring in the thumb may infect the common synovial cavity through a communication at its upper extremity.

In the former case a tender swelling forms along the thenar eminence and passes above the wrist on the radial side. When the common synovial cavity becomes invaded the hand becomes extremely painful, the fingers immobile, while the palm becomes swollen and tense, a secondary swelling appears for about an inch above the anterior annular ligament, and there is much œdema of the back of the hand. In superficial suppurations a cellulitis may occur in the forearm and even spread over the entire arm. When the deep tissues of the palm are involved suppuration is liable to occur amongst the tendons and muscles of the forearm; this may be associated with a superficial cellulitis.

In many cases of whitlow a simple lymphangitis exists—a leash of red streaks passing up the forearm from the focus of suppuration. In the larger lymphatics a thrombosis may occur, especially in those vessels converging on the axilla—appearing as hard knotted cords, which may eventually disappear or break down and suppurate at various points.

Even in the absence of a definite lymphangitis, the lymphatic glands may become enlarged and suppurate. The supra-condylar glands are involved in septic processes of the inner side of the hand, and I have been able to count as many as five of them in this position. From this area a further extension may occur to the axillary glands, leading to a painful enlargement or axillary abscess. In suppuration of the outer part of the hand a tenderness may occur over the bicipital groove due to inflammation of the glands along the course of the cephalic vein.

The rarer complications that may be mentioned are acute spreading gangrene, tetanus, pyæmia, and septicæmia.

#### Treatment.

One must mention prophylaxis: the care of a wound and its antiseptic treatment may entirely abolish whitlow. When inflammation has occurred, the whole finger must be carefully cleansed, spirit soap or lysol being efficient preparations. The nail should be trimmed closely, and any accumulation of dirt removed. The general surgical principles are to be followed in the early stages: Rest, fomentations four-hourly, and elevation—the arm being carried in a sling.

In the early stages passive congestion brought about by means of the elastic bandage applied to the arm for twenty out of the twenty-four hours may be relied on to cure a number of cases without operative treatment; while the elastic tourniquet, if carefully and correctly applied so as to cause venous congestion without pain or discomfort of itself relieves the pain and often enables the patient to sleep at night.

Should the inflammation fail to resolve, or continue to spread, further treatment of an operative nature must be carried out.

*The cuticular and sub-cuticular forms.*—After cleansing, the purulent blister is opened by means of the knife or scissors, and the whole of the separated epidermis removed close to its attachment to the normal skin. This simple operation is painless. The surface exposed is washed with some antiseptic solution, dried, dusted with boric powder, and a dry dressing applied. Should the dermis be involved, or a small subcutaneous wound be present, it is usually advisable to foment the part for twenty-four hours. Afterwards the region must be protected till the epidermis is sufficiently hard to stand the ordinary pressure.

*Subcutaneous* whitlow requires incising, should the part become brawny or if fluctuation is present. A longitudinal incision is made over the swelling and the skin divided, the abscess is finally opened by carefully deepening the incision or by means of the sinus forceps. Should the whole length of the finger be involved more than one incision is required, and these should be made if possible in the middle line on the palmar or dorsal surface or both.

*Thecal suppuration.*—In the case of the three fingers, index, middle, and ring, two incisions are required about half an inch long, the first situated over the distal part of the second phalanx and proximal part of the terminal. The second is made over the head of the metacarpal bone so as to open the sheath at its proximal end. By this means free drainage is given to the tendon sheath. The sheath should not be opened in its whole length; if this is done, the tendon stands out of its covering and usually sloughs.

The latter operations may be performed under local anæsthesia, the skin being infiltrated with a solution of eucaine and adrenalin. In cases of doubt as to how far the infection has spread it is better to give a general anæsthetic. A tourniquet having been applied to the arm, the finger is carefully incised. If pus is found in the subcutaneous tissue it is usually unnecessary to proceed further. If doubt is still felt as to the condition of the tendon sheath, it may be explored by means of a syringe and opened in the manner above described should it be infected. It is of great importance that the sheath be not opened if it has not been infected, since when the finger is laid open from end to end and the tendon exposed to septic material it frequently sloughs and the finger becomes almost useless.

In cases of suppuration of the distal portion of the finger an early incision may save periosteal infection and the death of the phalanx. Should the phalanx be necrosed it can be readily removed when separation has occurred, and it is usually advisable to wait for this to occur as its forcible removal is apt to lead to an opening in the tendon sheath, and may be followed by thecal or general suppuration.

In *thecal suppuration* of the thumb, an incision is made over the distal part of the first phalanx, a second above the wrist, to the outer side of the tendon of the flexor carpi radialis (the radial artery being protected, or in some cases divided). It may be necessary to make a third incision on the outer side of flexor brevis pollicis midway between the first two.

*Palmar suppuration.*—The little finger requires incision as for the other fingers. A second incision is made above the wrist to the inner side of the flexor carpi radialis and carefully deepened till the synovial sheath is reached. A third incision is required in the palm, and is made in the axis of the ring finger above the level of the superficial palmar arch.

In suppuration of the forearm an incision is made over any swelling present, and carefully opened by dissecting between the various structures till the pus is reached. A small tube or piece of gauze being inserted for drainage, while a counter opening may also be necessary.

In all these cases a general anæsthetic is given, the limb being rendered bloodless, and a careful dissection carried out so that no important structure is damaged. After operative treatment passive congestion accelerates the healing process, and often checks any further spread of the infection.

